# Actigraphic investigation of circadian rhythm functioning and activity levels in children with mucopolysaccharidosis type III (Sanfilippo syndrome)

**DOI:** 10.1186/s11689-015-9126-5

**Published:** 2015-09-01

**Authors:** Rachel A. Mumford, Louise V. Mahon, Simon Jones, Brian Bigger, Maria Canal, Dougal Julian Hare

**Affiliations:** Psychological Services, Alder Hey Children’s Hospital, Liverpool, UK; Manchester Mental Health & Social Care NHS Trust, Manchester, UK; School of Psychology, Cardiff University, Cardiff, UK; Genetic Medicine Unit, St Mary’s Hospital, Manchester, UK; Faculty of Medical and Human Sciences, University of Manchester, Manchester, UK; Faculty of Life Sciences, University of Manchester, Manchester, UK

**Keywords:** Mucopolysaccharidosis type III, Sanfilippo, Sleep, Circadian rhythms, Actigraphy

## Abstract

**Background:**

Sleep disturbance is part of the behavioural phenotype of the rare genetic condition mucopolysaccharidosis (MPS) type III. A growing body of evidence suggests that underlying disturbance in circadian rhythm functioning may explain sleep problems within the MPS III population.

**Methods:**

Actigraphic data were recorded in eight children with MPS III over 7–10 days and compared to age-matched typically developing controls. Parameters of circadian rhythmicity and activity levels across a 24-h period were analysed.

**Results:**

Statistically and clinically significant differences between the two groups were noted. Analysis indicated that children with MPS III showed significantly increased fragmentation of circadian rhythm and reduced stability with external cues (*zeitgebers*), compared to controls. Average times of activity onset and offset were indicative of a phase delayed sleep-wake cycle for some children in the MPS III group. Children with MPS III had significantly higher activity levels during the early morning hours (midnight–6 am) compared to controls.

**Conclusions:**

Results are consistent with previous research into MPS III and suggest that there is an impairment in circadian rhythm functioning in children with this condition. Implications for clinical practice and the management of sleep difficulties are discussed.

## Background

### Sleep and circadian rhythm functioning in intellectual and developmental disability

There is evidence that sleep disturbance and disorder occur more frequently in the intellectual and developmental disability (IDD) population [1, 2]. Prevalence estimates have been reported as high as 85 % [3, 4] although rates vary with age, level of disability and parental perceptions of sleep problems. Settling difficulties (increased sleep latency), frequent night waking, parasomnias, co-sleeping and daytime sleepiness are the most common difficulties described by parents [5, 6]. There is evidence that deficient or disrupted sleep can confer numerous disadvantages for the individual, including adverse effects on cognition, physical health and daytime behaviour [7, 8]. Poor parental psychological wellbeing is also associated with sleep disturbance in children with IDD [9, 10].

A number of explanations for this increased incidence of sleep disruption within the IDD population exist, including co-morbid physical health conditions, pain, sensory differences and environmental factors [11, 12]. Alongside these factors, a growing body of research has identified underlying circadian rhythm disruption as a further explanation of sleep problems in IDD. Circadian rhythms are internally generated physiological and behavioural cycles that occur across a 24-h period. They are influenced by internal biological factors and external environmental cues (*zeitgebers*) and include the sleep-wake cycle, hormone production and core body temperature regulation. Disturbance in circadian rhythm functioning has been reported in children and adults with autistic spectrum disorders [13–16], Smith-Magenis syndrome [17], Rett syndrome [18] and Angelman syndrome [19], amongst other IDD conditions. Specific circadian rhythm differences highlighted have included both phase advanced and delayed sleep syndrome, irregular sleep-wake patterns, increased fragmentation of rhythmicity and reduced stability or association with external *zeitgebers*.

Endogenous melatonin plays a role in the regulation of several bodily rhythms, including the sleep-wake cycle. Consequently, consistent with reports of sleep disturbance, abnormalities in levels of melatonin or in melatonin production have been identified in autism [20], Angelman syndrome [19] and Smith-Magenis syndrome [21]. Specifically in Smith-Magenis syndrome, a complete inversion of the circadian rhythm of melatonin release has been described and connected to the sleep disturbance and daytime behavioural difficulties observed in this condition [17, 21]. Accordingly, exogenous melatonin is often used as treatment for sleep disturbance for individuals with IDD [22, 23] although the potential value of behavioural sleep hygiene interventions is not dismissed [7].

### Sleep in MPS III

The mucopolysaccharide (MPS) disorders comprise a group of inherited metabolic diseases in which specific lysosomal enzymes are absent or deficient, causing cellular accumulation of glycosaminoglycans (GAGs). This accumulation impairs cellular function across organs [24, 25]. MPS III (Sanfilippo syndrome) is characterised by a deficiency in one of four enzymes involved in the metabolism of the glycosaminoglycan heparin sulphate. There are four corresponding subtypes (MPS III A, B, C and D) based on the specific enzyme alteration [26]. MPS III is the most common of the MPS disorders, with incidence estimated at 1 in 20, 000 live births [24]. It is characterised by a clinical course of three phases, involving progressive cognitive and physical deterioration [24, 26]. In the first phase (1–4 years), developmental delay becomes increasingly apparent after an initial period of typical development. The second phase begins between 3 to 4 years and is marked by an increasing loss of function, severe sleep disturbance and behavioural difficulties, including hyperactivity and aggression. The third phase (10+ years) is characterised by progressive loss of skills, increased motor difficulties, seizures and respiratory complications. Life span is significantly reduced, with death often reported in the second or third decade [26]. Current clinical treatment focuses on symptom management although potential therapies that will attempt to modify the progression of the disease (enzyme replacement therapy, gene therapy and haematopoietic stem cell transplantation) are under development [27].

Sleep disturbance has consistently been reported in MPS III, with incidence rates of 87–92 % [28–31] and is therefore described as part of the behavioural phenotype of the condition [32]. Parent report studies have identified a broad spectrum of sleep difficulties, including difficulty initiating sleep, frequent night/early morning waking, disruptive behaviour at night (e.g. wandering, singing) and daytime sleepiness [28, 29, 31, 33]. Clinicians working with families affected by MPS III also report these as the main presenting sleep-related difficulties [34]. Objective measurement of sleep in MPS III has been carried out in two studies. Actigraphic analysis undertaken with eight individuals with MPS III subtype A or B revealed significantly longer sleep onset latencies and increased daytime sleep in the MPS III group compared to controls [33]. There were also trends towards increased night waking and reduced sleep efficiency in the MPS III group. Older age was associated with longer sleep onset latency and diminished sleep efficiency for individuals with MPS III. Parent questionnaire responses indicated significantly greater sleep disturbance across all domains in the patient group compared to controls. One study [35] has used polysomnography (PSG) and electroencephalogram (EEG) to investigate sleep in a small sample of individuals aged 7–20 years with MPS III subtype A. Compared to controls, there was reduced nocturnal sleep duration, REM sleep and slow wave sleep, alongside greater daytime sleep in the MPS III group. Older individuals with MPS III were reported to show an irregular and fragmented sleep-wake pattern, with no identifiable circadian rhythm.

There is growing evidence that sleep disturbance in MPS III may be explained by an abnormality in the diurnal release of melatonin. Levels of melatonin production in MPS III have been shown to be significantly lower at night and higher during the day [36]. Additionally, Mahon et al. [33] reported no significant variation in melatonin concentration across three time points within a 24-h period in MPS III patients. This suggests an alteration in circadian melatonin production, given that levels can be differentiated across time in typically developing children. These findings directly correspond with subjective and objective reports of the nature of sleep disturbance observed in MPS III (i.e. difficulty initiating sleep at night, night waking and daytime sleepiness) and parallel research into sleep disturbance and an inversion of the circadian rhythm of melatonin release observed in Smith-Magenis syndrome [17, 21]. Alterations in circadian functioning have also been demonstrated in murine models of MPS III [37, 38], with MPS IIIB mice showing weaker circadian rhythm cycles, increased activity levels during rest phases and a marked late peak of activity within the light/dark cycle compared to age-matched control mice.

There is limited research into treatment for sleep disturbance in MPS III. Given the abnormalities in diurnal melatonin concentration, exogenous melatonin is often the pharmacological treatment of choice, with some level of effectiveness reported by parents in approximately 70 % of individuals with MPS III [31]. In this study [31], 60 % of families also reported some degree of success with behavioural strategies although only 37 % of the overall sample had tried a behavioural intervention. There is therefore undoubtedly scope for further research into the acceptability and effectiveness of behavioural intervention for sleep in MPS III. Finding effective treatment is critical given that sleep disturbance has a major impact on individuals with MPS III and their families. Parents report disrupted sleep patterns for the whole family [29], and both parents and clinicians have described associations between sleep difficulties and increased daytime challenging behaviour in children with MPS III [31,34]. Sleep disturbance places extra strain on parents who are already coming to terms with their child’s diagnosis and its emotional, social and financial burdens. Previous research has indicated that parents of children with MPS III experience clinically significant levels of depression and anxiety [39, 40]. There are, therefore, significant clinical implications for families if an increased understanding of sleep and circadian rhythmicity in MPS III results in tailored, effective interventions.

### Aims and hypotheses

This study formed part of a wider investigation into MPS III undertaken at the University of Manchester in conjunction with the Department of Genetic Medicine, St Mary’s Hospital, Manchester. This paper presents additional analysis of the data published by Mahon et al. [33]. The primary aim was to specifically examine parameters of circadian rhythm functioning related to the activity/rest cycle in children with MPS III through the use of objective actigraphic assessment. It was hypothesised that children with MPS III would exhibit a significantly different pattern of circadian functioning, compared to typically developing (TD) controls.

## Methods

The National Research Ethics Service North West-Lancaster Research Ethics Committee approved the research protocol.

### Participants

Ten families of children with MPS III expressed an interest in participating; however, two families were subsequently withdrawn from the study due to technical problems with actigraphic recording and one child’s additional health complications. Consequently, eight children with MPS III took part in the study: five males and three females, aged 2–15 years (mean = 9 years 3 months, SD = 4.86). Individual demographic information is presented in Table 1. All children had a diagnosis of MPS III subtype A or B. Diagnosis was verified through urine/specific-enzyme analysis, and the presence of sleep disturbance was not a prerequisite to participation. Exclusion criteria were as follows: (i) concurrent involvement in an enzyme replacement study, (ii) previous bone marrow transplant, (iii) serious disease affecting another organ, or (iv) child considered near the end of life. The two eldest children with MPS III subtype A had epilepsy. Older children were prescribed more medications, including medication for sleep (melatonin, chloral hydrate, zopiclone). Given the established effect of exogenous melatonin on circadian rhythmicity [41], this treatment was discontinued 2 weeks prior to data collection. All other medication was unchanged throughout data collection. Previous interventions for sleep had been attempted for four children with MPS III (including melatonin, herbal medication and behavioural techniques) with limited effectiveness as reported by parents. Eight children, four males and four females, aged 3–15 years (mean = 8 years 7 months, SD = 4.85) formed an age-matched (within 1 year), TD control group. These children were not taking any medication and did not have developmental disability, neurological disorder, brain injury, sleep disorder or mental health difficulties.

**Table 1:** Demographic information of MPS III participants

Participant ID	Sex	Age (years)	MPS III subtype	Ethnicity	Current medication/intervention (effectiveness^a^)	Previous sleep intervention (effectiveness^a^)
MPS1	Male	2	B	Pakistani	None	None
MPS2	Male	4	A	White Polish	None	None
MPS3	Male	5	B	White British	None	Behavioural advice (parents had already tried techniques)
MPS4	Male	10	B	Pakistani	Melatonin^b^ (good for settling) Loperamide	Herbal medicine (no effect after first week)
MPS5	Female	10	B	Pakistani	Walking (helpful) Risperidone (no effect)	None
MPS6	Female	11	A	White British	Gonapeptyl	None
MPS7	Male	14	A	White British	Chloral hydrate, zopiclone (short-term effects only), levetiracetam, ibuprofen, hyoscine	Melatonin (short-term effect only) Behavioural techniques (no effect)
MPS8	Female	15	A	White British	Zopiclone, clonazepam, midazolam, sodium valproate, Senokot, Movicol, omeprazole, glycopyrrolate, morphine, paracetamol	Melatonin (no effect) Temazepam (initial effect but discontinued due to distress/tearfulness)

^a^Effectiveness of intervention for sleep as reported by parents

^b^Melatonin treatment ceased 2 weeks prior to data collection

### Procedure

All eligible families of a child with MPS III under the care of St Mary’s Hospital, Manchester, or registered with the MPS Society UK were sent a recruitment letter providing brief details of the project and asking them to make initial contact with the researchers. The control group was comprised of children of colleagues from the University of Manchester or local NHS Trust. Interested families were given participant information sheets, and a home visit was subsequently conducted to obtain consent, explain actigraphy and collect demographic data. Written informed consent was obtained from a parent of each child. In the TD control group, participants aged 14 to 15 years gave their informed consent and assent was also obtained from those aged 6 to 13 years. Participants received a gift voucher for taking part.

### Actigraphy

Actigraphy allows for the collection of naturalistic data over an extended time period and has been shown to correlate highly with PSG [42]. Either a Cambridge Neurotechnology AW4 or Respironics Actiwatch 2 actigraph was used to obtain data. These are equivalent models of actigraph produced under different names allowing for comparability of data. All children wore the actigraph on their non-dominant wrist continuously for 7–10 days as recommended by previous research [43]. Actigraphs were only removed when washing or swimming. Data was sampled across 15-s epochs. To ensure comparability of data across subjects and to limit any seasonal impact on circadian functioning, all data was collected during a standard school week within a 6-month period.

### Circadian rhythm and activity parameters

Actigraph data was downloaded and analysed using Actiware version 5.5 to provide the following established non-parametric indices of rhythmicity [44]:

i. L5 and M10 onset specify the average time of the start of the least active 5-h period (L5) and the most active 10-h period (M10) across a circadian cycle and provide an indication of the extent to which an individual’s circadian cycle is coordinated with a normal 24-h cycle.
ii. Relative amplitude is derived from the normalised difference between the most active 10-h period and least active 5-h period in an average 24-h pattern and indicates the quantity of activity, with a range of 0–1 (higher values represent a greater divergence between most and least active phases).
iii. Intra-daily variability indicates fragmentation of an individual’s rhythm, through assessing the frequency and extent of transitions between rest and activity, and is derived as a ratio of the mean squares of the differences between all successive hours and the mean squares of difference within the grand mean. Derived scores range from 0–2, with higher values indicative of increased fragmentation.
iv. Inter-daily stability specifies the invariability of 24-h rhythm between days and is the 24-h value from a chi-square periodogram, normalised for number of data points. It provides an indication of the strength of the linkage of the circadian rhythm to external zeitgebers that are deemed to be stable within the period of recording (e.g. daylight), with a range of 0–1 (higher values indicate greater stability). The parameter is derived by examining the regularity of the pattern of data points across each day of recording.
v. Periodicity specifies the time of the peak correlation of the ‘best fit’ of any given rhythm within the parameters of the expected circadian cycle of 24 h ± 15 min.

In addition, overall levels of activity were measured across each of the four quadrants of a 24-h period (i.e. midnight–6 am, 6 am–12 pm, 12 pm–6 pm and 6 pm–midnight). This data allowed for analysis between groups of potential differences in activity at particular time points during the day. Total activity counts were also generated for each day and averaged across the recording period.

### Statistical analysis

SPSS version 20 was utilised for further analysis of actigraphic data using non-parametric statistics (Mann-Whitney *U*, Spearman’s Rank correlation). Two-tailed tests were adopted throughout, with a significance level of 0.05. It was necessary to exclude actigraph data from one night for several participants due to illness or non-compliance; however, there was a minimum of seven nights’ data for all participants.

## Results

### Circadian rhythm analysis

**Fig. 1:**
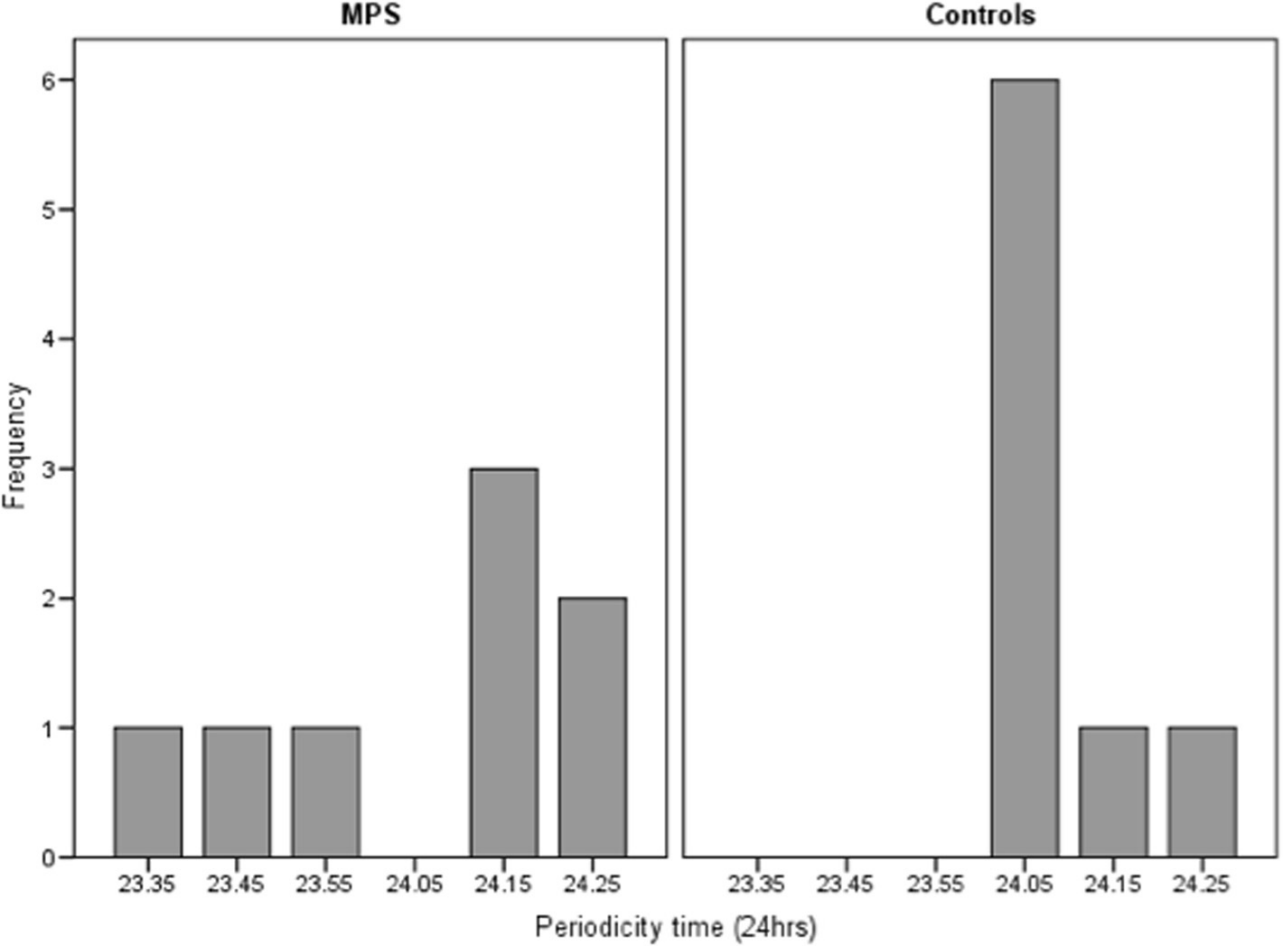
Periodicity (time of peak correlation) for MPS III and control group

**Fig. 2:**
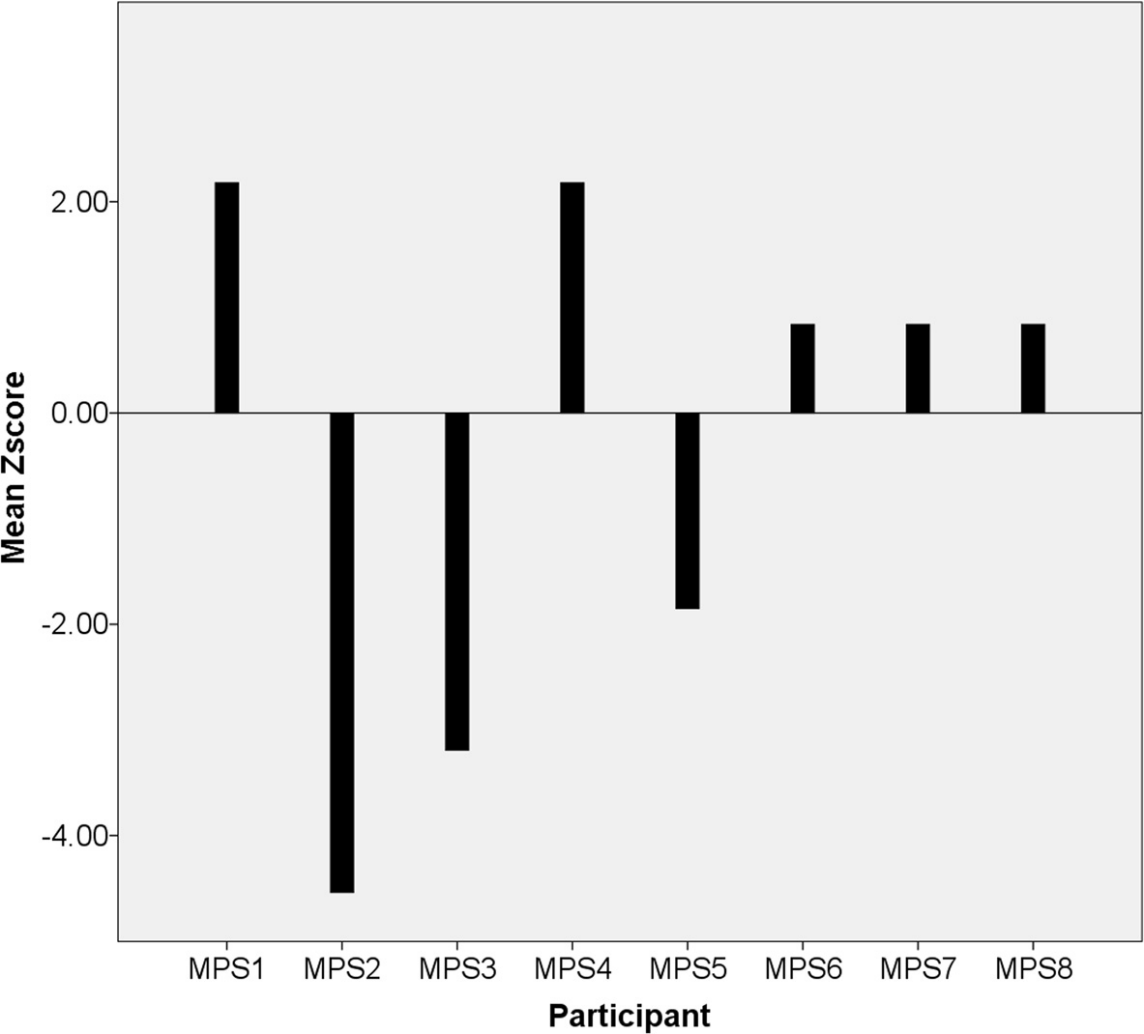
Periodicity z-scores for MPS III group compared to controls

Non-parametric circadian rhythm analysis [44] was performed on the actigraph data obtained from all participants. Data was averaged over the recording period, as indicated by previous research. Table 2 presents descriptive statistics of circadian rhythm parameters for the MPS III and control groups, alongside inferential analyses. As can be seen, mean intra-daily variability was significantly greater in the MPS III group compared to controls (*U* = 6.5, *z* = −2.68, *p* = 0.007, *r* = 0.67 (large effect)), indicating increased fragmentation of rhythm across the recording period. Additionally, mean inter-daily stability was significantly lower in the MPS III group than in the control group (*U* = 13.0, *z* = −1.99, *p* = 0.046, *r* = 0.5 (large effect)), showing less stability of rhythm across days in relation to external *zeitgebers*. Despite lower mean relative amplitude in the MPS III group, this was not found to be significantly different to controls (*U* = 16.0, *z* = −1.68, *p* = 0.093, *r* = 0.42 (medium-large effect)). Mean periodicity (peak correlation of circadian rhythm) did not differ between groups (*U* = 29.5, *z* = −0.27, *p* = 0.785, *r* = 0.06), but the standard deviation for periodicity was much greater in the MPS III group, with some children showing either a slightly phase advanced or delayed circadian rhythm cycle (see Fig. 1). Further analysis via calculation of z-scores indicated that half of the MPS III group demonstrated significantly different periodicity scores to controls. Two children showed significantly phase advanced circadian rhythms (23½ hours, *z* = −4.54 and 23¾ hours, *z* = −3.19), whereas two children demonstrated significantly phase delayed circadian rhythms (24½ hours, *z* = 2.18 for both children). Figure 2 presents individual MPS III group periodicity z-scores compared to controls. The wide variation within the MPS III group accounts for the non-significance of mean periodicity group comparisons.

**Table 2:** Comparison of circadian rhythm parameters for MPS III and control groups

	MPS III (*n* = 8)		Controls (*n* = 8)			
	Mean	Median	Mean	Median	*U*	*p*
	(SD)	(IQR)	(SD)	(IQR)	(2 tailed)	
Relative amplitude	0.85	0.94	0.95	0.95	16.0	0.93
	(0.19)	(0.13)	(0.02)	(0.04)		
Intra-daily variability	0.88	0.86	0.71	0.73	6.5	0.007**
	(0.12)	(0.22)	(0.08)	(0.14)		
Inter-daily stability	0.50	0.50	0.62	0.62	13.0	0.046*
	(0.12)	(0.20)	(0.08)	(0.14)		
Periodicity^a^	1446.25	1455	1448.75	1445	29.5	0.785
	(18.85)	(35)	(7.44)	(7.50)		

^a^24-h time converted to minutes

*p < 0.05; **p < 0.01

The data for L5 onset (least active 5-h period) and M10 onset (most active 10-h period) indicated wide variability in both phases within the MPS III group. There was a bimodal L5 onset of 11 pm and 2 am in the MPS III group (range = 9 pm to 3 am), compared to a mode of 11 pm for L5 onset in the control group (range = 10 pm to 1 am). The mode for M10 onset was 12 pm in the MPS III group (range = 8 am to 12 pm) and 9 am for controls (range = 8 am to 12 pm). Taken together, these modal figures illustrated a later onset of wakefulness and activity for children with MPS III, combined with wider variation in the onset of their least active period. For three children with MPS III, these parameters were indicative of phase delay in the sleep-wake cycle (L5 = 2 am, M10 = 11 am; L5 = 3 am, M10 = 12 pm; and L5 = 2 am, M10 = 12 pm).

### Activity analysis

Activity levels were averaged over the recording period for each quadrant of a 24-h period (midnight–6 am, 6 am–12 pm, 12 pm–6 pm and 6 pm–midnight). Individual data for the MPS III group is presented in Table 3, with group comparisons in Table 4. There was wide variation in activity levels amongst the children with MPS III. Five out of eight children had higher activity counts towards the end of the day in the fourth quadrant (6 pm–midnight), compared with their level of activity during the morning (6 am–12 pm). This is further suggestive of a phase delayed sleep-wake cycle. The youngest three children, and in particular those in the second phase of the disorder (aged 4 and 5 years), displayed the highest overall levels of activity. The three children with MPS III who had the lowest overall activity levels, which included the eldest two children (14 and 15 years), showed the least discrepancy in level of activity across the 24-h period. An attenuated trajectory of activity for children with MPS III is evident, with little observable difference in activity levels between the second (6 am–12 pm) and fourth (6 pm–12 pm) time quadrants.

**Table 3:** Activity levels in children with MPS III averaged over recording period

Participant ID (age)								
Time quadrant	MPS1	MPS2	MPS3	MPS4	MPS5	MPS6	MPS7	MPS8
	(2)	(4)	(5)	(10)	(10)	(11)	(14)	(15)
Midnight–6 am	9.33	6.59	6.47	2.72	7.87	4.25	7.08	27.14
6 am–12 pm	42.18	125.32	172.36	15.10	23.12	78.29	21.01	35.89
12 pm–6 pm	116.45	153.93	198.21	21.09	112.32	145.79	49.11	53.77
6 pm–Midnight	128.86	61.75	62.07	23.15	102.29	52.39	24.71	54.25
Total daily activity	296.82	347.59	439.11	62.05	245.61	280.72	101.92	171.05

**Table 4:** Comparison of activity levels for MPS III and control groups

	MPS III (*n* = 8)		Controls (*n* = 8)			
	Mean	Median	Mean	Median	U	*p*
	(SD)	(IQR)	(SD)	(IQR)	(2 tailed)	
Midnight–6 am	8.93	6.84	3.79	4.03	11.0	0.027*
	(7.64)	(4.16)	(2.12)	(4.01)		
6 am–12 pm	64.16	39.03	100.52	104.72	10.0	0.037*^a^
	(57.17)	(92.02)	(23.38)	(42.86)		
12 pm–6 pm	106.33	114.39	142.99	158.19	20.0	0.208
	(60.60)	(101.62)	(37.42)	(66.59)		
6 pm–Midnight	63.68	58.00	56.58	59.34	30.0	0.834
	(36.10)	(66.61)	(19.31)	(27.99)		
Total daily activity	243.12	263.16	304.51	311.27	21.0	0.248
	(126.23)	(215.69)	(55.38)	(103.24)		

^a^Inferential analysis after removal of extreme outlier

*p < 0.05

Group comparisons (see Table 4) indicated a significantly greater mean level of activity for children with MPS III within the first time quadrant (midnight–6 am), compared to controls (*U* = 11.0, *z* = −2.21, *p* = 0.027, *r* = 0.55 (large effect)). There were no statistically significant differences between groups in the mean activity scores for the third quadrant (12 pm–6 pm; *U* = 20.0, *z* = −1.26, *p* = 0.208, *r* = 0.31 (medium effect)) or fourth quadrant (6 pm–midnight; *U* = 30.0, *z* = −0.21, *p* = 0.834, *r* = 0.05). Although descriptive analysis suggested that activity levels were lower in the MPS III group compared to controls for the second time quadrant (6 am–12 pm), this difference did not reach statistical significance (*U* = 18.0, *z* = −1.47, *p* = 0.141, *r* = 0.38 (medium-large effect)). However, following removal of one extreme score within the MPS III group, a statistically significant difference between groups was detected (*U* = 10.0, *z* = −2.08, *p* = 0.037, *r* = 0.52 (large effect)). There was no significant difference between groups in mean total activity scores across the recording period (*U* = 21.0, *z* = −1.16, *p* = 0.248, *r* = 0.29 (small-medium effect)).

Spearman’s rank correlational analyses were conducted for both circadian rhythm and activity parameters. Despite some observable differences in activity parameters across age in the MPS III group, age was not significantly correlated with any variables in either the MPS III or control group. There was a trend towards a negative relationship between age and relative amplitude in the MPS III group (*r* = −0.70, *p* = 0.056) indicating, in line with descriptive statistics, that older children had less differentiation between the most and least active phases across the 24-h period.

## Discussion

As hypothesised, actigraphic assessment of circadian rhythm and activity parameters revealed clinically and statistically significant differences between children with MPS III and TD controls. Children with MPS III demonstrated significantly increased variability and fragmentation of circadian rhythm (intra-daily variability) across a 7–10-day period, with less stability of rhythm across days in relation to external *zeitgebers* (reduced inter-daily stability). There was a marginal trend towards lower relative amplitude in the MPS III group, suggesting that there was less differentiation between the most and least active phases over the 24-h period compared to TD controls. For children with MPS III, individual periodicity scores and times of activity onset and offset were indicative of incidences of both phase delayed and phase advanced sleep-wake cycles although phase delay was more common. There was also greater variability of activity onset and offset times within the MPS III group, which is consistent with results for intra-daily variability and inter-daily stability parameters.

Descriptive analysis of activity level patterns showed a shallower rhythm of activity in the MPS III group, which was attenuated in its rise and fall. This finding corresponds with a trend towards lower relative amplitude (i.e. less differentiation between periods of activity and rest) in individuals with MPS III. Children with MPS III demonstrated significantly higher levels of activity in the early morning hours (midnight–6 am) compared to controls. Descriptive analysis suggested lower activity levels between 6 am and 12 pm in the MPS III group and, after the removal of one outlier, this difference was found to be statistically significant. This is consistent with a later onset of the most active 10-h period in the MPS III group and provides evidence of a later onset of wakefulness and activity, which is suggestive of phase delayed circadian rhythm functioning.

Taken together, both circadian rhythm and activity parameters were indicative of disrupted circadian rhythm functioning in children with MPS III, and this may offer some explanation of the significant sleep disturbance reported within this population [28–31, 33]. Circadian functioning was quantitatively different to TD controls and this finding is consistent with previous research utilising PSG, which reported irregular sleep-wake patterns in individuals with MPS III [35]. With PSG methodology some individuals with MPS III demonstrated no identifiable circadian rhythm of the sleep-wake cycle; however, this finding was not replicated in the present study. This inconsistency may be accounted for by the fact that the PSG study also included adults with MPS III in their sample, who would have been at a later stage of the disease. An increased fragmentation of sleep and circadian functioning has previously been associated with older individuals with MPS III [33, 35]. Accordingly, in the current study, there was a marginal trend towards reduced differentiation between most and least active phases and greater disruption in patterns of activity levels in older children with MPS III. This provides further tentative evidence that sleep and circadian rhythm disturbance may be related to disease progression.

The finding that levels of activity were significantly higher in the MPS III group in the early morning (midnight–6 am) extends previous reports of increased nocturnal wakefulness in MPS III [35]. These bouts of activity may likely relate to episodes of night behaviours and parasomnias as reported by parents [31, 33]. Furthermore, the reduction in activity levels between the hours of 6 am and 12 pm and a later onset of wakefulness in the MPS III group may fit with parent and clinician reports of daytime sleepiness [31, 34]. Both these findings, along with non-parametric indices of rhythmicity, are suggestive of phase delayed circadian functioning in MPS III, although further investigation with a larger sample is required to confirm this. Descriptive analysis of activity data also demonstrated that children in the second phase of MPS III (age 4 and 5 years) had the greatest levels of activity throughout the day. This corresponds with clinical reports of hyperactivity during this period [24, 26, 28].

Evidence of impairment in circadian rhythm functioning in MPS III is consistent with reports of an abnormality in endogenous melatonin concentrations [33, 36]. Melatonin secretion typically coincides with the onset of darkness, peaks during the night and is inhibited in the early morning with light onset. Paradoxically, melatonin production in MPS III has been demonstrated to be significantly lower at night and higher during the day [36] corresponding with sleep onset difficulties and night waking and daytime sleepiness, respectively. In the present study, this could potentially offer an explanation for higher activity levels between the hours of midnight–6 am and reduced activity between 6 am–12 pm in children with MPS III. An abnormality of melatonin production is also consistent with significantly lower inter-daily stability within the MPS III group, given the relationship between melatonin secretion and external *zeitgebers* (e.g. light). As a consequence, both lines of research point towards a potential intrinsic alteration in circadian clock functioning in MPS III, which is also consistent with murine models of the condition [37]. The identification of circadian rhythm disturbance in MPS III is also in line with previous research into sleep and circadian functioning in various IDD conditions [14–19]. Being ‘out of sync’ with environmental *zeitgebers* and the sleep-wake pattern of others is likely to have a major impact on quality of life for the individual and those who care for them. Learning opportunities or valued activities may be hindered [7], and expressions of challenging behaviour may be increased [3, 45], placing further strain on family functioning. It is, therefore, of central importance to address sleep-wake difficulties in individuals with MPS III.

### Limitations

Given the rarity of MPS III, the sample size was small and thus variability within the data may have limited detection of statistically significant results. Accordingly, some effect sizes for non-significant results were indicative of difference between groups. There were observable clinical differences in circadian rhythm functioning across age (and consequently, disease stage) in the MPS III group, with increased disruption in circadian rhythm generally in older children. However, the small sample size limited the statistical power to detect significant relationships based on age/stage of disease and thus prevents any firm conclusions from being drawn. It was also not possible to make any comparisons between MPS III subtypes A and B, which could be a critical factor given that subtype A is considered more severe [46] and both the eldest children were diagnosed with subtype A. The eldest two children with MPS III also had epilepsy, which has been shown to relate to sleep difficulties [47], but it was not possible to investigate or control for the potential impact of this co-morbid condition.

Although exogenous melatonin was withdrawn prior to actigraphic monitoring, some children were prescribed further hypnotic medications. It is possible, therefore, that circadian rhythm parameters may have been further disrupted for these children without medication use although the effect may have only been minimal as there is evidence that these medications have little effect on sleep architecture [48]. More likely is the potential impact of these medications on measurement of activity across the 24-h period, based on a side effect profile of increased daytime lethargy and somnolence [49]. This may partially explain the overall lower levels of activity in the two eldest children with MPS III taking these medications, although other circadian parameters were nonetheless indicative of disrupted rhythmicity. Medication side effects is one of the challenges posed by research into rare conditions [50], and it may be particularly difficult to overcome in the MPS III population, given the common co-morbidity of physical health complaints.

Although some research has indicated that actigraphy is less accurate in discriminating between sleep-wake states in populations where sleep is fragmented [51], it generally correlates highly with PSG data [42]. The results of the present study largely correspond with findings obtained with PSG in an MPS III population [35]. Actigraphy has been used to differentiate sleep-wake states in individuals with physical disabilities to an acceptable degree of reliability with PSG, albeit slightly lower than for individuals without physical disability [52]. Despite this, it may have been beneficial to include measurement of any restrictions on physical activity in the MPS III group (e.g. time spent in a wheelchair, use of restraint), given that this is a common factor in MPS III. There are further potentially relevant factors that could have been recorded, including school attendance, behavioural difficulties, parental management strategies and family composition.

### Future research

International, multi-centre research into circadian functioning and sleep in MPS III would allow for validation of the present findings in a larger sample and would enable comparisons between MPS III subtypes and across stages of disease progression. It might also permit the recruitment of unaffected siblings to act as a more appropriate control group. Longitudinal analysis with actigraphy may also allow for exploration into potential differences in ultradian rhythm functioning, which, given the variability within the MPS III sample, may warrant investigation. Simultaneous measurement of melatonin and cortisol concentration levels across the 24-h period would also enhance understanding of circadian clock functioning and its relative contribution to sleep disturbance within this population. There is evidence that intrinsic circadian clock functioning has an influence on respiratory systems [53], and a high prevalence of sleep-disordered breathing has been reported across all MPS conditions [54]. Therefore, further work could usefully examine whether there is a relationship between nocturnal breathing difficulties and circadian rhythm functioning in MPS III.

The relationship between sleep problems and daytime challenging behaviour in IDD populations has been identified in a number of studies [3, 5, 16, 45]. Given the high reported rates of behavioural difficulties in MPS III [26, 39], combining actigraphy with measurement of daytime behaviour would allow for objective investigation of this potential relationship. Additionally, further research may examine the relative contributions and effectiveness of behavioural and pharmacological interventions for sleep problems in MPS III.

### Clinical implications

An increased understanding of circadian rhythm functioning and sleep patterns of children with MPS III has significant implications for individuals and families. ‘Sleep problems’ in IDD populations are by no means equivalent issues and should not be treated so. Estimating individual circadian functioning is therefore critical in order to guide appropriate treatment choices, together with the timing of any treatment administration (e.g. melatonin, bright light therapy) [55]. Employing actigraphy in clinical practice with MPS III populations would aid assessment and enable interventions to be targeted to individual difficulties. It would also provide a baseline of sleep and circadian rhythm functioning in a condition involving progressive developmental decline, which may allow for effective evaluation of new treatments targeting disease progression [27].

Enhancing parental understanding of their child’s circadian rhythm and sleep disruption may inherently relieve some of the associated anxiety and stress regarding sleep; although the impact of caring for a child with sleep difficulties is by no means dismissed. By providing an explanation for the sleep problem, any negative perceptions that parents may hold in relation to their child’s sleep or parenting abilities could potentially be altered. Using actigraphy would enable these individual sleep patterns to be identified and explained to parents, allowing for implementation of daytime and bedtime routines that fit with the individual child’s circadian rhythm. Evidence supports the use of behavioural intervention for sleep problems in children with IDD [7, 56]; therefore, there should be routine screening for sleep difficulties in children with MPS III, comprehensive assessment of the nature of sleep disturbance and implementation of subsequent tailored interventions incorporating behavioural strategies. Based on parent and clinician report, exogenous melatonin has been suggested as the most effective pharmacological treatment for sleep problems in MPS III [31, 34]; however, its limited, short-term effects in some cases may be explained by an increased disruption of circadian rhythm functioning. Alternative treatments, such as light therapy and pharmacological agents acting upon the endogenous melatonin system, are considered for the circadian rhythm disorder, Smith-Magenis syndrome [21, 57] and may be worth an investigation as treatment options in MPS III.

## Conclusions

This study provides further evidence for the notion of an impairment in circadian rhythm functioning in children with MPS III. Further research is required to investigate this difference longitudinally, across MPS III subtypes and in relation to the circadian rhythm of endogenous melatonin and cortisol systems. Given the high prevalence of sleep problems in MPS III and the negative impact of childhood sleep disturbance on individual and family functioning, these findings are particularly relevant for clinical management of sleep in MPS III. It remains imperative to attempt to ascertain the underlying aetiology of sleep and circadian rhythm disturbance, in order that appropriate behavioural and pharmacological interventions can be targeted for the individual.
